# A study on the reproducibility of cephalometric landmarks when undertaking a three-dimensional (3D) cephalometric analysis

**DOI:** 10.4317/medoral.17721

**Published:** 2012-02-09

**Authors:** Natalia Zamora, José M. Llamas, Rosa Cibrián, José L. Gandia, Vanessa Paredes

**Affiliations:** 1 PhD, MSc, Orthodontist, Assistant Professor, Department of Orthodontics, Faculty of Medicine and Dentistry, University of Valencia, Valencia, Spain; 2 PhD, Orthodontist, Associate Professor and Director of Postgraduate Orthodontics Masters Course, Department of Orthodontics, Faculty of Medicine and Dentistry, University of Seville, Seville, Spain; 3 PhD, Associate Professor, Department of Physiology, Faculty of Medicine and Dentistry, University Valencia, Valencia, Spain; 4 PhD, Orthodontist, Associate Professor and Department Chair of Postgraduate Orthodontics Masters Course, Department of Orthodontics, Faculty of Medicine and Dentistry, University of Valencia, Valencia, Spain; 5 PhD, Orthodontist and PhD Assistant Professor, Department of Orthodontics, Faculty of Medicine and Dentistry, University of Valencia, Valencia, Spain

## Abstract

Objectives: Cone Beam Computerized Tomography (CBCT) allows the possibility of modifying some of the diagnostic tools used in orthodontics, such as cephalometry. The first step must be to study the characteristics of these devices in terms of accuracy and reliability of the most commonly used landmarks. The aims were 1- To assess intra and inter-observer reliability in the location of anatomical landmarks belonging to hard tissues of the skull in images taken with a CBCT device, 2- To determine which of those landmarks are more vs. less reliable and 3- To introduce planes of reference so as to create cephalometric analyses appropriated to the 3D reality. Study design: Fifteen patients who had a CBCT (i-CAT®) as a diagnostic register were selected. To assess the reproducibility on landmark location and the differences in the measurements of two observers at different times, 41 landmarks were defined on the three spatial axes (X,Y,Z) and located. 3.690 measurements were taken and, as each determination has 3 coordinates, 11.070 data were processed with SPSS® statistical package. To discover the reproducibility of the method on landmark location, an ANOVA was undertaken using two variation factors: time (t1, t2 and t3) and observer (Ob1 and Ob2) for each axis (X, Y and Z) and landmark. The order of the CBCT scans submitted to the observers (Ob1, Ob2) at t1, t2, and t3, were different and randomly allocated. Multiple comparisons were undertaken using the Bonferroni test. The intra- and inter-examiner ICC´s were calculated. Results: Intra- and inter-examiner reliability was high, both being ICC ≥ 0.99, with the best frequency on axis Z. Conclusions: The most reliable landmarks were: Nasion, Sella, Basion, left Porion, point A, anterior nasal spine, Pogonion, Gnathion, Menton, frontozygomatic sutures, first lower molars and upper and lower incisors. Those with less reliability were the supraorbitals, right zygion and posterior nasal spine.

** Key words:**Cone Beam Computed Tomography, cephalometry, landmark, orthodontics, reliability.

## Introduction

Modern Cone Beam Computerized Tomography (CBCT) systems applied to oral and maxillofacial regions provide a helpful diagnostic tool in orthodontics (1). This technology eliminated the errors of the conventional radiographs (magnification, image distortion, superimposition of anatomical structures) and the drawbacks that medical computed tomography (CT) presented (high economic costs, high radiation doses) (2). Several authors have compared these radiation doses between all kinds of devices (3-5), all of them concluding that conventional systems (panoramic radiography and lateral radiography) continue to emit the lowest radiation doses, followed by CBCT and lastly conventional CT. Nevertheless, it has been observed that several orthodontic patients need not only panoramic radiography and lateral radiography, but also other additional radiographs; such as a posteroanterior radiography to assess asymmetries, periapical series in periodontal problems, occlusal radiographs or even a magnetic resonance in order to assess the temporomandibular articulation. The sum of the effective doses of all these additional radiographs exceeded the effective dose of the CBCT. Therefore, in these cases, the use of the CBCT would be recommended instead of undertaking all those above mentioned radiographs separately (5,6). CBCT also allow the possibility of modifying some of the diagnostic tools used in orthodontics, such as cephalometry. The first step to undertake cephalometric studies on CBCTs, must be to study the characteristics and limitations of these devices in terms of accuracy and reliability of the most commonly used landmarks. Despite the fact that many studies have evaluated the accuracy and reliability both in CTs (7-10) and CBCTs (11-23) many of them evaluate linear distances. This methodology presents the limitation that in case the measurement is seen to be lacking in accuracy, we cannot determine which of the two points forming the line is the inaccurate one (7). Training and familiarization with the location of cephalometric landmarks is essential because landmark identification errors are considered a major source of cephalometric errors (15). Moreover, landmarks must be located from whichever viewpoint as exact points and with a unique anatomical location (8) and must be defined for each of the 3D spatial planes as coordinates (20). Once the easier points to locate are known, we can introduce planes of reference for a 3D cephalometric analysis.

The aims of this study were; 1- To assess intra and inter-observer reliability in the location of anatomical landmarks belonging to hard tissues of the skull in images taken with a CBCT device, 2- To determine which of those landmarks are more vs. less reliable and 3- To introduce planes of reference so as to create cephalometric analyses appropriated to the 3D reality.

## Material and Methods

A study approved by the ethical committee of the Clinical University Hospital of the University of Valencia was undertaken. Fifteen (n=15) patients were selected of between 8 and 27 years of age (mean age: 15.27 ± 5.34 (SD) years old), 73.4% females and 26.6% males. The CBCTs had been taken because some of these patients were scheduled for orthognathic surgery, while the other ones presented with impacted maxillary canines.

Scans of these patients were undertaken using the CBCT i-CAT® (Imaging Sciences International, Hatfield, Pa) equipment. This apparatus generates a total of 306 slices with an image matrix size of 575x575. It was set at medium quality and high resolution. The portrait mode field of view (FOV) was employed, which gathers data in extended FOV mode and includes the entire head (170 mm x 230 mm) with a scanning time of 8.9 seconds. The voxel size was 0.4 x 0.4 x 0.4 mm. Tube voltage is 120 kVp and its intensity 23.87 mAs. The gross data and the slices obtained were imported to Beta NemoStudio® software (Software Nemotec SL, Madrid, Spain) where the post-processing of the medical images, their conversion into DICOM (Digital Imaging and Communications in Medicine) format, and 3D reconstructions were undertaken.

**Figure 1:**
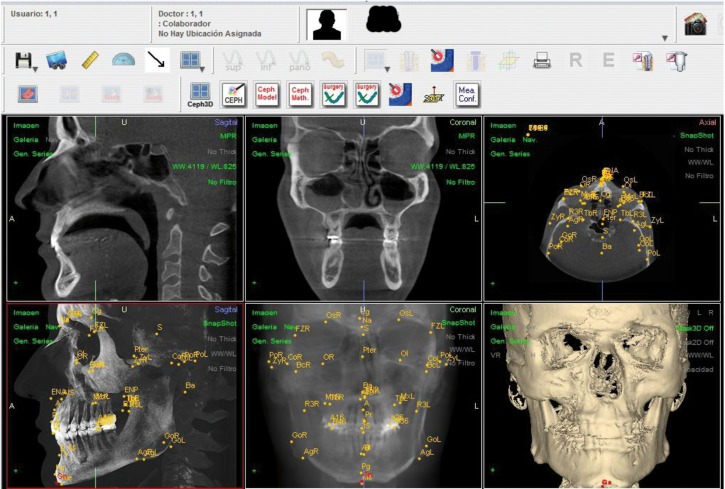
Sagittal, coronal, axial and 3D reconstruction windows.

41 landmarks belonging to the hard tissues of the skull were defined. The landmarks chosen are commonly used in orthodontics for locating craniofacial structures, and they represent landmarks that clinicians are familiarized to recognise and locate without difficulty ( Table 1). The software automatically determined the origin of the coordinates (0,0,0) at the right anterior lower corner of the cube that contained the 3D image and 3 axes were defined: X parallel to the right-left direction, Y in posterior-anterior direction and Z in the upper-lower direction. Once the coordinate system had been standardized, spatial positions of each landmark were represented as numerical values (in mm) on each axis. In order to fine-tune the location of points, the software permitted the visualization of two windows for the sagittal view and another two for the coronal or frontal view, which, moreover, could be enlarged to full screen using the zoom. By doing so, we were able to simultaneously observe the right and left sides or have two projections of the same side open for the sagittal view, or for the coronal view (MPR= multiplanar reconstruction, Raysum= X-ray projection, MIP=maximum intensity projection) (Fig. 1). To locate each landmark, the slice of the most appropriate plane was selected before fitting it onto the other planes for greater accuracy.

**Table 1:**
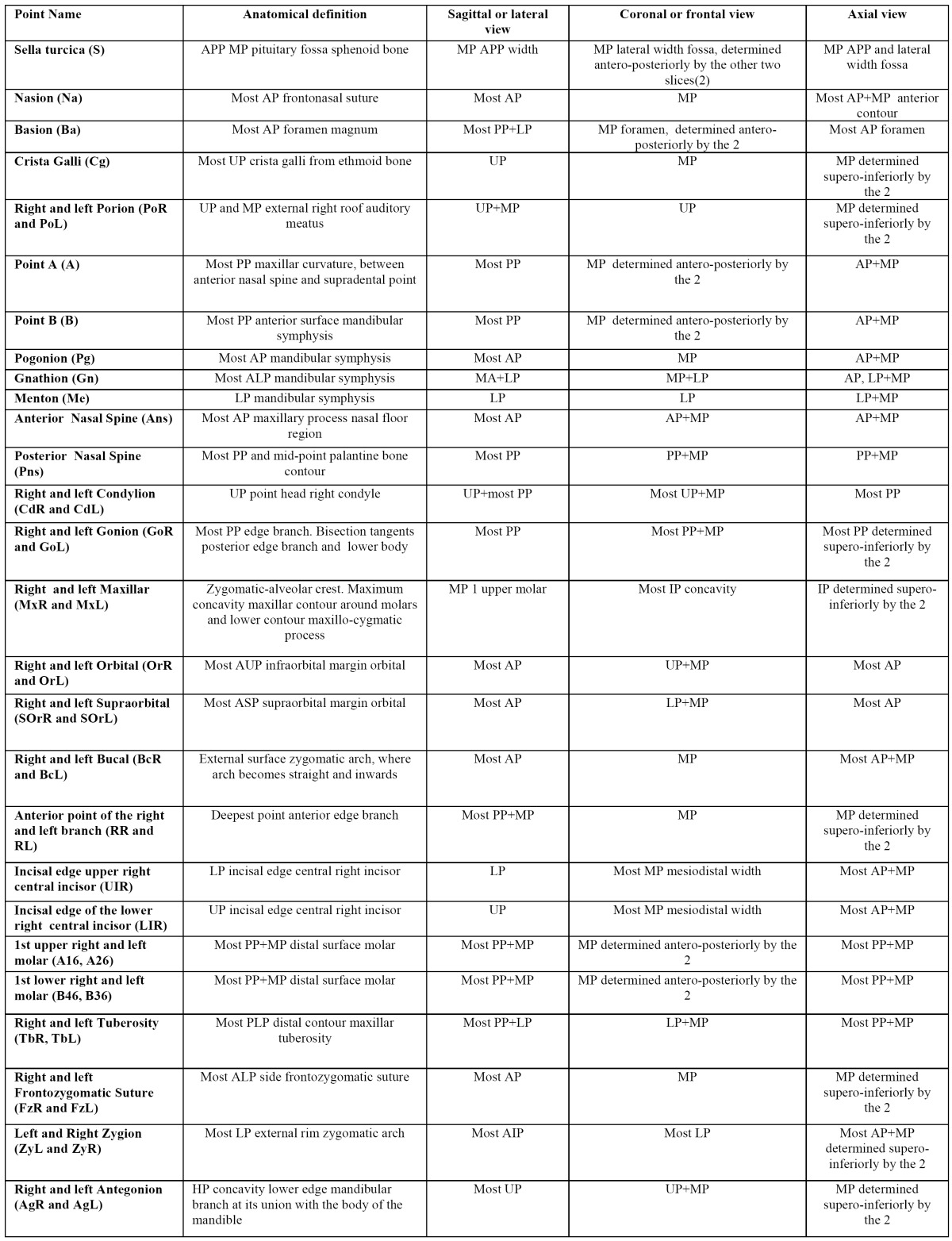
Definition of the three spatial planes of the 41 points used in this study. Anteroposterior point(APP), Mid point (MP), Posterior point(PP), Lowest Point(LP), Upper Point(UP), Anterlower Point (ALP), Anteriorupper.Point(AUP), Posteriorlower Point(PLP), Highest Point(HP), Inner Point (IP).

To evaluate reproducibility and intra- and inter-observer error, two (2) previously trained and qualified observers in the location of cephalometric landmarks, both with six years of experience/background in orthodontics, repeated the measurements on three occasions at intervals of one week. 3.690 measurements were taken and, as each determination has 3 coordinates, 11.070 data were processed with SPSS® statistical package 17.0 for Windows (IBM Corporation, Sommers, NY).

To discover the reproducibility of the method on landmark location, an ANOVA was undertaken using two variation factors: time (t1, t2 and t3) and observer (Ob1 and Ob2) for each axis (X, Y and Z) and landmark. The order of the CBCT scans submitted to the observers (Ob1, Ob2) at t1, t2, and t3, were different and randomly allocated. Multiple comparisons were undertaken using the Bonferroni test. The intra- and inter-examiner ICC´s were calculated. In our study, we decided to use the ICC since the measurements were numeric and not categorical, in which case we would have used the Cohen’s kappa test. Besides, the ICC had been used by other authors reviewed in our work and this allows us to compare the results in a very similar way. Reproducibility errors were found for each landmark. A regression line was determined for comparing the values between Ob1 and Ob2, estimating the slope, ordinate at origin and the Pearson correlation coefficient. So that the measurements of both observers could be considered equivalent, the confidence interval of the slope had to contain the 1 and the confidence interval of the ordinate at origin, the 0.The difference between ratios was determined estimating the confidence interval of that difference. Differences were considered significant for p<0.05 and confidence intervals were determined at 95%.

## Results

We analysed the 1.845 determinations made by each observer (Ob) considering the value of each axis independently, so as to find out whether the reproducibility of locating the different landmarks was associated with one or other axis of coordinates.

-Intra and inter-observer time variability 

Prior to calculating the reliability of each landmark, an evaluation of the raw data was performed. The global mean and the stan-dard deviation of each observer (Ob1, 2) was calculated in Time (1,2,3) in the X,Y,Z planes. A statistically significant difference was found for Ob 1 for axis Y in t1 compared with t2 and t3 (p=0.006 between t1-t2; and p=0.008 between t1-t3). There was no statistically significant difference for Ob1 between t2-t3 and for Ob2 on any of the axes.

The ICC determined was ≥ 0.99 for all the axes in intra-observer measurements and ≥ 0.99 in inter-observer measurements, with the highest values corresponding to axis Z (ICC > 0.996).

-Reliability and measuring method errors

**Figure 2:**
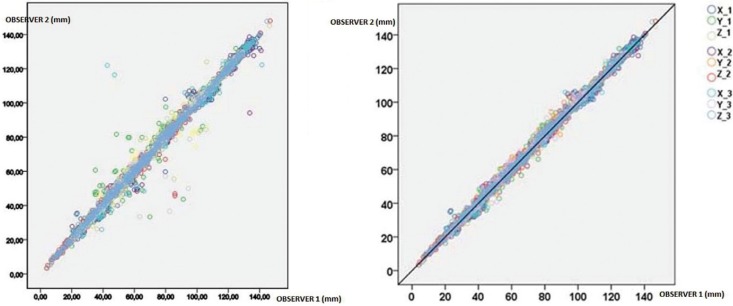
Single graphic with Values (mm) of the 1845 determinations with standard deviation (SD) of less than 0.5 mm and maximum of 2 mm, undertaken by Observer 1 versus the corresponding 1845 determinations undertaken by Observer 2, for each axis.

The value of the coordinate of each landmark has been represented in a single graphic. The number of values represented amounts to 5.535. We have represented t-1, t-2 and t-3 of Ob 1(abscissae) versus t-1, t-2 and t-3 of Ob 2 (ordinates) (Fig. 2).

The points on the bisection were taken into account for determining the method sensitivity error. The points outside the bisection corresponded to the landmarks with the highest errors between observers.

For each landmark 6 determinations were made. The observations that diverged from the bisection could be a consequence either for being one determination that diverges from the other five, or because, even though the intra-observer measurements were similar, there were differences between observers. The determination which deviated more than 10 mm from the mean and those where the mean of the values from the three determinations undertaken by Ob 1 diverged more than 10 mm from the mean of those of Ob 2, for the same point in the same patient, were considered as wrong determinations.

With these criteria there were 61 wrong determinations (Fig. 2). This means an error of 0.55% CI95% [0.54% - 0.56%]. Ob1 presented 0.45% CI95% [0.44% - 0.66%] and Ob 2, 0.65% CI95% [0.64% - 0.66%]. They did not have the same distribution on the three axes. 13 (0.11%) correspond at X, 38 (0.34%) at Y and 10 (0.09%) at Z ( Table 2).

**Table 2:**
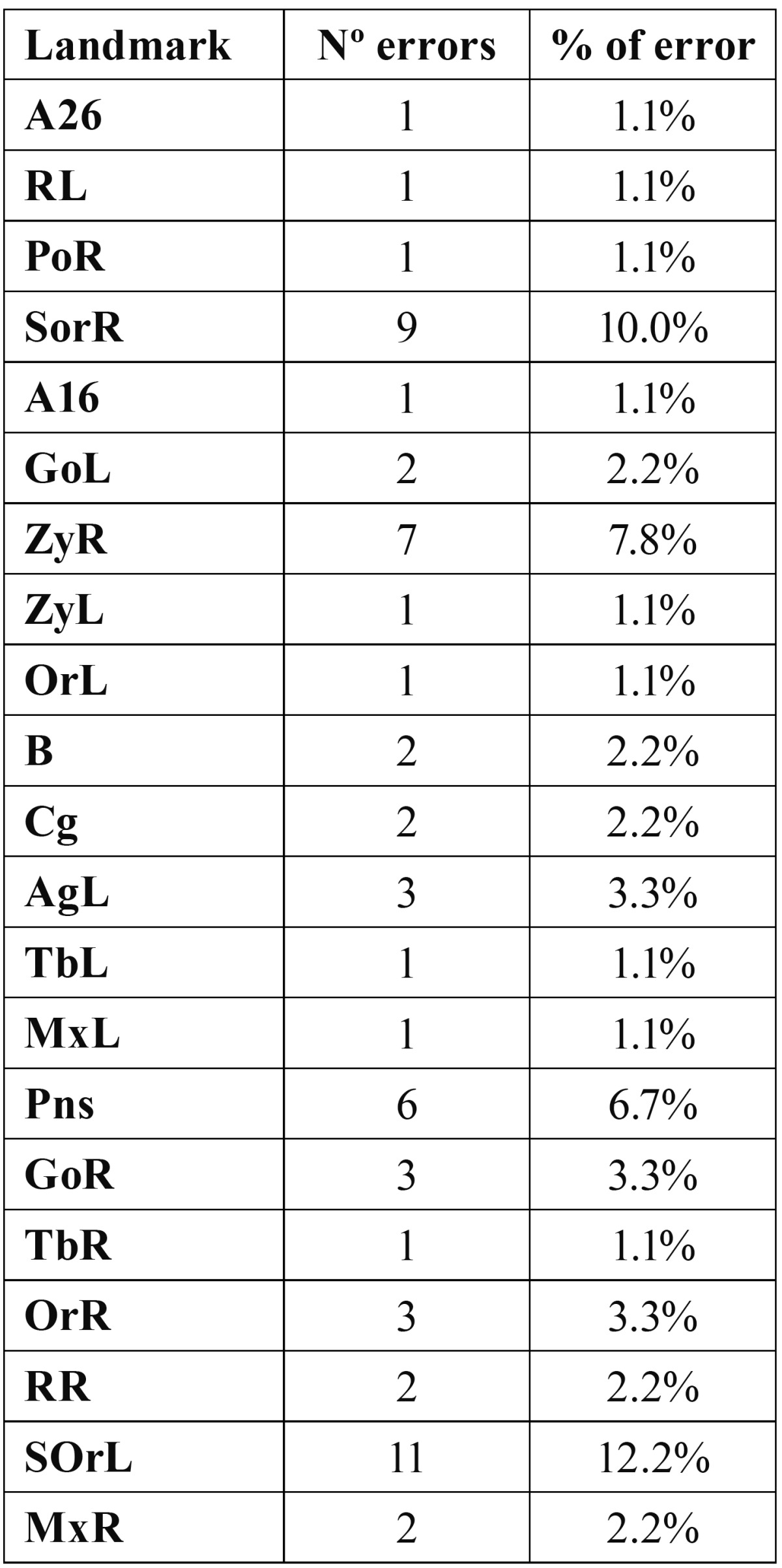
Landmarks with high error. Each anatomical point has been measured 90 times, as the 3 measurements have been undertaken by each observer on 15 patients. Dark and light grey represent the landmarks with the highest error.

In evaluating the data, the decision was made to remove from the data set any identified landmarks that were technical errors. We decided to do it in that way because large outliers were due to technical errors in the use of the software and not due to the misunderstanding of the landmark definition or inability to locate the landmark in the 3-D image. With those criteria, the 61 wrong determinations were eliminated, in order to determine the method sensitivity error. The adjustment line of these points has a Pearson correlation coefficient of 0.998 with a slope of 0.999 CI95% [0.997, 1.001] and an ordinate at origin of 0.007 CI95% [-0.124, 0.137], showing that the measurements of both observers are perfectly comparable.

Standard deviation (SD) of each coordinate (X, Y, Z) was analysed for each landmark measured on each patient. According to these results, accuracy was quite similar on all 3 axes ( Table 3).

**Table 3:**
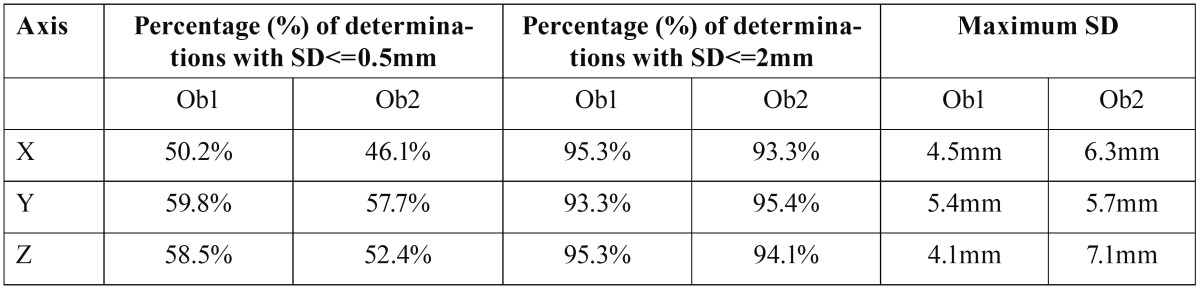
Maximum standard deviations (SD) and percentage (%) of determinations with standard deviations (SD) of less than 0.5 mm and 2 mm, for each observer (Ob1, Ob2) and each coordinate (X, Y, Z).

The average SD of all the landmarks was 1.0 mm, which corresponds to an average relative error of 1.3%. 74% of the measurements on X axis, 76.5% on Y axis and 69.7% on Z axis were below 1.5% of error. The 95% CIs showed only a statistically significant difference between the percentage of Y axis [74.1%, 78.8%] and that of X axis [67.1%, 72.2%].

The average SD that corresponded to each anatomical landmark was also determined and classified by areas of interest ( Table 4).

**Table 4:**
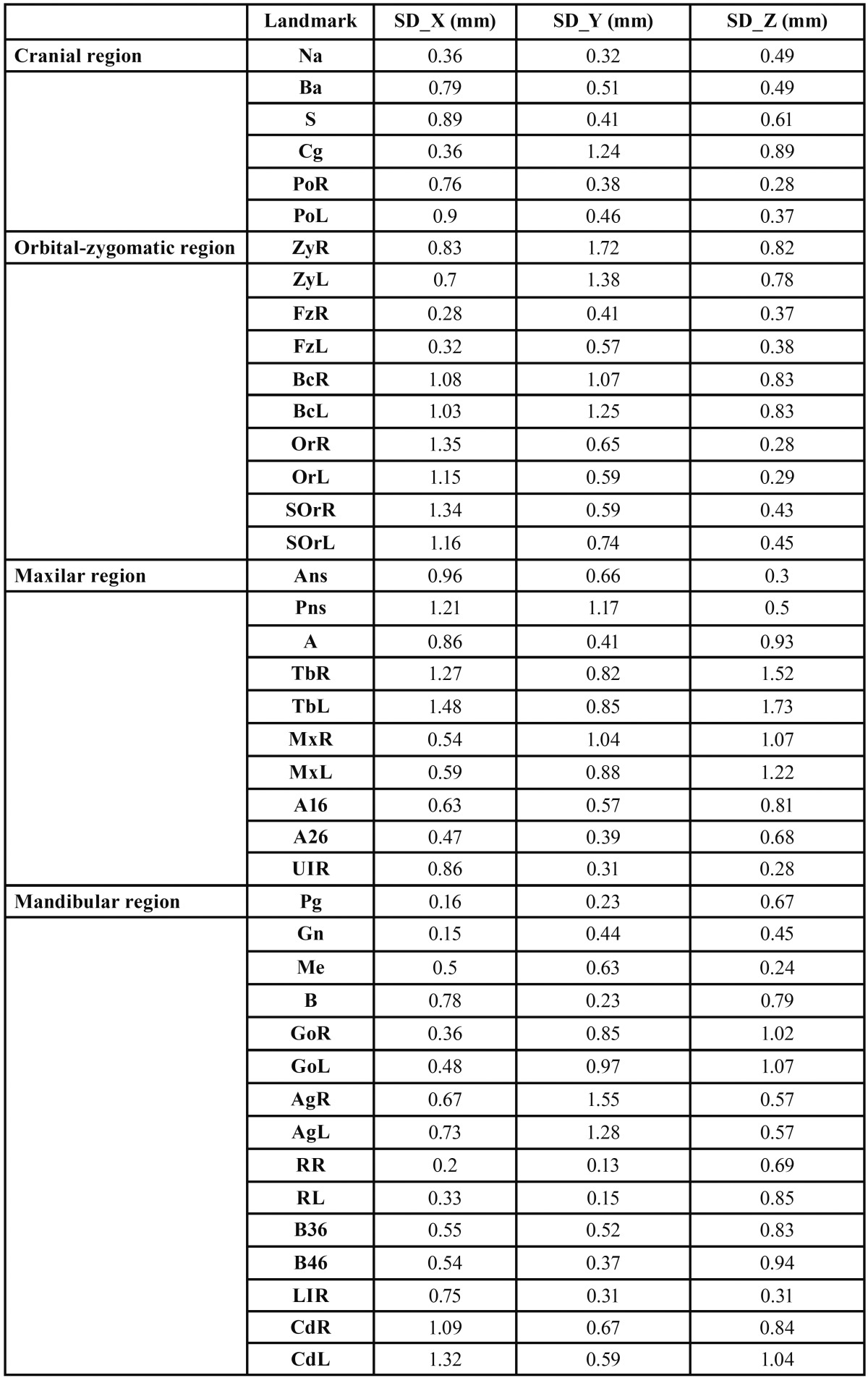
Average standard deviation (SD) in millimeters (mm) corresponding to each anatomic landmark. Those represented in shading had SD on all axes (X, Y, Z) below the average value (equal to 1.0 mm) and present less measurement error.

## Discussion

In the present study the reproducibility in the location of the different landmarks commonly used for orthodontic diagnosis when making a cephalometric tracing has been determined. Of all the studies reviewed, our work is the one with more CBCTs and cephalometric points used. In order to carry out this study, records of patients who had already undergone a CBCT for various reasons were used. Other studies use dry skulls to undertake the measurements, as patient irradiation is not justified unless strictly necessary. However, and despite the fact that certain studies state that soft tissues distort the measurements, it is necessary to undertake them with all the tissues included, in order to be able to check more accurately and to simulate the clinical situation(15,23). The drawback of carrying out a study of these characteristics is that irradiating actual patients for this purpose is not justified and therefore the sample in our study was restricted to only fifteen patients who had required CBCTs for surgical reasons or for the presence of impacted teeth.

In this study each of the 41 landmarks was also described and defined for each of the coordinates of the space (X, Y, Z). As recommended by Oliveira et al. (20), this definition in the three spatial planes facilitates the true landmark position. The problem of some studies is that they do not give a detailed definition of the landmarks in each of the spatial planes as we do (15,16). All the landmarks of this study belonged to anatomical structures. In CT and CBCT, superimpositions of structures are not generated and, therefore, the points based on them have little interest in this type of records (6).

In the present study, two observers determined in three occasions the location of 41 landmarks and 11.070 data were processed unlike the study of Schlicher et al.(16) where nine examiners made the measurements but only in one occasion (5472 data), analyzing only half of our measurements. In the overall intra-observer time variability a statistically significant difference was only found for Ob 1 for axis Y in t1. This result can be interpreted both as a learning process that takes place in the location of the landmarks in this axis in the last two measurements, or as a fortuitous situation associated with the measuring process. As in our study, Park et al.(9) and Kumar et al.(24) did not find inter-examiner statistically significant differences between the 19 landmarks or the 14 landmarks measured, respectively. Schlicher et al.(16) did not find either significant improvement in the identification of the 32 landmarks measured. The results coincide with ours but with a smaller number of points to locate (19, 14 and 32 respectively compared with the 41 points of our work). Although intraobserver landmark identification errors are generally lower than interobserver errors in Lagravère´s study (15) and ours not completely coincide since they showed that intra- and interobserver ICCs were similar. The high intra-observer and inter-observer ICCs found indicate high degree of reliability in locating landmarks, following the observers’ prior learning process. Our results coincide with those of Oliveira et al. (20) who found an ICC ≥0.9 for 85% of the intra-observer measurements and for 65.5% of the inter-observer measurements. They obtained the best ICC, as we did, on the Z axis (93.3%). In their study only 1% of the intra-observer and 3% of the inter-observer measurements had an ICC< 0.45. Our results agree partially with those of Lagravère et al.(22) which found an ICC > 0.97 for the intra-observer and an ICC> 0.92 for the inter-observer measurements; with the results of another study taken by the same authors (15) with an ICC>0.99 for the intra-observer and inter-observer measurements; and with those of Park et al.(9) who concluded that the reliability of all their landmarks was high, showing a good location on the three axes. As Schlicher et al. (16) calculated the average coordinates of each landmark (calculated for the two observers in the three occasions) but a gold standard was not included in the study.

In our study, of the 41 landmarks measured, half of them, 20 did not present errors in their determination in any of the 90 measurements taken (Na, S, Ba, PoL, A, Ans, Pg, Gn, Me, FzR, FzL, B36, B46 UIR, LIR, BcR, BcL, CdR, CdL, AgR). This suggests that these landmarks could be used safely when establishing their position. However, of the 21 remaining landmarks, SOrL, SOrR, ZyR and Pns, with more than 6 errors in their determination, would correspond with landmarks that should not be used as a basis for devising cephalometries or for which greater training would be necessary before using them in an analysis. As Schlicher et al. (16) stated, familiarity and anatomy could be responsible for the poorer performance in locating these landmarks. Those landmarks that presented the lower standard deviation (SD) on all the axes below the average value (1.0 mm) were Na, S, Ba, PoR, PoL, A, Ans, RR, RL, Pg, B, Gn, Me, FzR, FzL, A16, A26, B36, B46, UIR, LIR. If we consider the points which not only have not presented any error in their determination but have the least standard deviation (SD) value, then Na, S, Ba, PoL, A, Ans, FzR, FzL, Pg, Me, Gn, B36, B46, UIR, LIR appear to be the most reliable ones. These can be considered as reliable landmarks for being used in 3D cephalometric analyses.

If we analyse them per areas of interest, the cranial region showed the greatest reliability. Clinicians are accustomed to identify these landmarks in conventional cephalometry, especially Na, S and Ba, so this may explain their high reliability. High reliability of the frontozygomatic sutures (FzR, FzL) in the orbital-zygomatic region was also found. The landmarks with a greater margin of error were the supraorbitals (SOrR, SOrL). This may be due to the difficulty of locating them on those CBCTs that do not include the complete cranium. In the maxillary region, the landmarks of greatest reliability were the first upper molars (A16, A26), the upper incisor (UIR), the anterior nasal spine (Ans) and point A. Landmarks such as the maxillaris (MxR, MxL) or the retromolar tuberosities (TbR, TbL) were those with the greatest margin of error. The difficulty in locating these landmarks may be caused by the lack of practice at identifying them, because they are not usually used in conventional lateral cephalometrics. In the mandibular region, Gnathion (Gn), Menton (Me), Pogonion (Pg) and point B obtained the highest reliability. However, the condyles (CdR, CdL), gonions (GoR, GoL) or antegonials (AgR, AgL) had lower reliability.

As in our study, Lagravère et al. (15) found, in general, mean differences between intra-observer measurements of less than 1mm. In their study, those landmarks that were between 1-2 mm of error were: OrL, S, Ba, Ans, Pns in the X plane, GoR, GoL, PoL and Pns in the Y plane and point B and LIR in the Z plane. On the other hand, the mean differences between intra-observer measurements were, in general higher than 1mm, this results being different to those found in our study. They found that the less reliable landmarks (with > 2mm of error) were: OrR, OrL, PoR, PoL, CdR, CdL in the X plane, GoR, GoL and Ans in the Y plane; and GoR, GoL and LIR in the Z plane. In our study we also found low reliability for Pns, OrR, OrL, CdR, CdL in the X plane, Pns in the Y plane and GoR, GoL in the Z plane (although in our results the SD of these landmarks is always < 2mm). Our results did not totally coincide with those of Oliveira et al. (20) In their study, the two landmarks that presented low reliability were: Y coordinates of the right and left mandibular ramus and Z coordinate of the right and left condylion. We found low reliability in the right condylion at the X coordinate. However, our results coincide with those of Muramatsu et al. (10) who evaluated the reliability of 19 landmarks in images taken with CT, observing that the Basion had the lowest confidence area ellipse on all planes, which indicated great reliability. Other authors (16) found S point to be the most reliable landmark, as we did, and PoR and OrR the most unreliable ones.

In general, in our study we found that landmark location using CBCT has less than 1.5% of error. This indicates high reliability in the location of all the landmarks and, therefore, is of great interest for clinical application in 3D cephalometric analyses. Nevertheless, we must not forget that introducing a certain cephalometric analysis cannot be based only on its reproducibility, but also on its clinical significance and other factors.

Current 3D records are very useful, especially to accurately locate bilateral and transversal points that are better observed from the coronal plane. In this way it is possible to study and analyse, in greater depth and reliability, dentofacial asymmetries, problems on the transversal level of the maxillary bone structures, as well as the size of the structures that are involved in the skull.

When analysing the results, greater reliability is to be expected on the sagittal plane as clinicians are trained in locating landmarks in conventional 2D cranial lateral teleradiography. However, with a properly calibration and training, observers can locate, with high reliability, the landmarks on each of the three planes of the space. In general, landmark location in 3D requires greater time than in 2D; firstly because prior training is necessary to familiarize with the different slices presented, and secondly because a first location on one of the planes and then a plotting on the other two is required for accuracy (12). As this location differs from the 2D conventional radiography location and as many of our patients are asked to have a CBCT as a diagnostic tool, it has been established that with correct instruction and with adequate learning, clinicians should locate properly these landmarks directly on CBCTs, obtaining much more diagnostic information about the different relationships between the different craniofacial structures of the patient.

**Figure 3:**
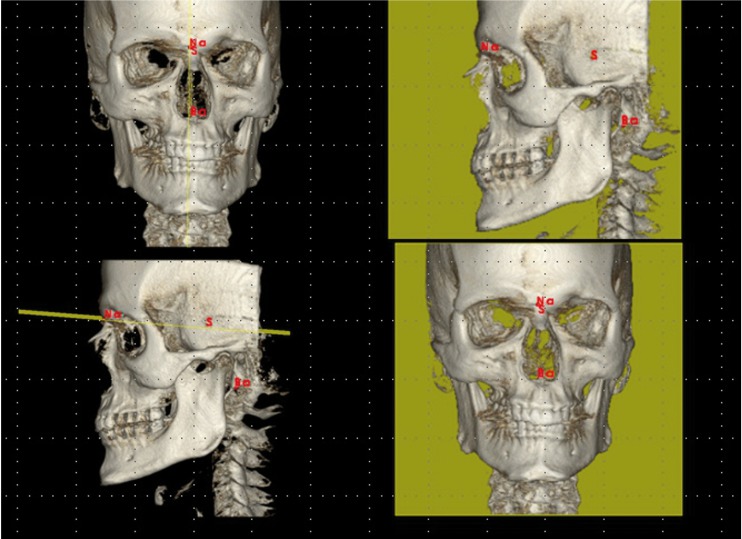
Planes of reference: Mid-sagittal plane (XZ): defined by the points Na, S, Ba; Horizontal or transversal plane (XY); Coronal or frontal plane (YZ).

One of the new things presented in this paper we have not seen in others is that based on the reliability of the landmarks studied, we have also defined three planes of reference. In line with this criterion of greater reliability, various anatomical landmarks could be chosen as a basis for defining these three planes of reference on the three spatial axes (Fig. 3).

• Mid-sagittal plane (XZ): anterior-posterior vertical plane that divides the body in two halves (right and left portions), defined by the points Na, S, Ba, points of high reliability and of easy location.

• Horizontal or transverse plane (XY): Horizontal plane perpendicular to the median sagittal plane that divides the body into upper and lower halves. We can obtain it and make it pass through each point that we choose, for example through Na.

• Coronal or frontal plane (YZ): vertical plane perpendicular to the two previous ones that goes from one side of the body to another dividing it into two parts (anterior and posterior). It cuts through the mid-sagittal plane in the middle.
